# Histone H3 Lysine 9 Methyltransferase DIM5 Is Required for the Development and Virulence of *Botrytis cinerea*

**DOI:** 10.3389/fmicb.2016.01289

**Published:** 2016-08-22

**Authors:** Xiaoli Zhang, Xinqiang Liu, Yanli Zhao, Jiasen Cheng, Jiatao Xie, Yanping Fu, Daohong Jiang, Tao Chen

**Affiliations:** ^1^State Key Laboratory of Agricultural Microbiology, Huazhong Agricultural UniversityWuhan, China; ^2^Provincial Key Lab of Plant Pathology of Hubei Province, College of Plant Science and Technology, Huazhong Agricultural UniversityWuhan, China

**Keywords:** *Botrytis cinerea*, histone H3 lysine 9 methyltransferase, H3K9 trimethylation, BcDIM5, virulence, development

## Abstract

Histone methylation is widely present in animals, plants and fungi, and the methylation modification of histone H3 has important biological functions. Methylation of Lys9 of histone H3 (H3K9) has been proven to regulate chromatin structure, gene silencing, transcriptional activation, plant metabolism, and other processes. In this work, we investigated the functions of a H3K9 methyltransferase gene *BcDIM5* in *Botrytis cinerea*, which contains a PreSET domain, a SET domain and a PostSET domain. Characterization of *BcDIM5* knockout transformants showed that the hyphal growth rate and production of conidiophores and sclerotia were significantly reduced, while complementary transformation of *BcDIM5* could restore the phenotypes to the levels of wild type. Pathogenicity assays revealed that *BcDIM5* was essential for full virulence of *B. cinerea. BcDIM5* knockout transformants exhibited decreased virulence, down-regulated expression of some pathogenic genes and drastically decreased H3K9 trimethylation level. However, knockout transformants of other two genes heterochromatin protein 1 (HP1) *BcHP1* and DNA methyltransferase (DIM2) *BcDIM2* did not exhibit significant change in the growth phenotype and virulence compared with the wild type. Our results indicate that H3K9 methyltransferase BcDIM5 is required for H3K9 trimethylation to regulate the development and virulence of *B. cinerea*.

## Introduction

Genetic information of eukaryotes is stored as chromatin, which consists of genomic DNA, histones, and a wide array of chromosomal proteins. In eukaryotes, DNA is wrapped around histones, which are subjected to a variety of covalent modifications, such as methylation, acetylation, phosphorylation, and ubiquitylation (Widom, 1998). Histone methylation mainly occurs on the side chains of lysines and arginines. Lysines may be mono-, di-, or tri-methylated, whereas arginines may be mono-, symmetrically, or asymmetrically di-methylated (Bedford and Clarke, 2009; Lan and Shi, 2009). Lysine methylation is highly selective, and K4 and K9 of histone H3 are the best characterized sites (Liu et al., 2010). In general, lysine 9 of histone H3 (H3K9) is associated with transcriptionally inactive heterochromatin, and is a well-conserved epigenetic mark for heterochromatin formation and transcriptional silencing, while H3K4 methylation is associated with transcriptionally active euchromatin (Berger, 2007).

The first histone lysine methyltransferase to be identified was SUV39H1, which targets H3K9 (Rea et al., 2000). Strikingly, all of the histone lysine methyltransferases that methylate N-terminal lysines contain a so-called SET domain that harbors the enzymatic activity. *Neurospora crassa* DIM5 can specifically tri-methylate H3K9 (Xiao et al., 2003). Orthologs of SUV39H1, which are named as Clr4 in yeast (Nakayama et al., 2001), Su(var)3-9 in Drosophila (Tschiersch et al., 1994), and Suv39h1 in mice (O'Carroll et al., 2000), are the major heterochromatic H3K9 methyltransferases and play a dominant role in pericentric heterochromatin formation.

*Botrytis cinerea* (teleomorph: *Botryotinia fuckeliana*) causes severe loss in more than 200 crop species worldwide. It is most destructive on the mature or senescent tissues of dicotyledonous hosts and ornamentals (Williamson et al., 2007). *B. cinerea* is a typical necrotroph fungus, whose infection strategies include killing of the host cells and feeding on the dead tissues by secreting cell wall degrading enzymes, and toxic metabolites that induce cell death prior to the invasion of hyphae (Siewers et al., 2005; Choquer et al., 2007). Two polyketide synthases (*BcPKS6* and *BcPKS9*) are required for phytotoxin botcinic acid biosynthesis and virulence (Dalmais et al., 2011). Two velvet-like genes (*BcVeA* and *BcVELB*) are involved in the regulation of fungal development, oxidative stress response, and virulence in *B. cinerea* (Yang et al., 2013). Presilphiperfolan-8 beta-alpha synthase *BcBOT2* is responsible for the first step of botrydial synthesis (Wang et al., 2009), whereas *BcBOT1* is a P450 monooxygenase that acts in the later step of biosynthesis (Siewers et al., 2005). Methylation of histone H3 is required for the normal development of *N. crassa* (Adhvaryu et al., 2005), and methylation of lysine H3K9 is a mark that primes the formation of heterochromatin and a critical chromatin landmark for genome stability (Rivera et al., 2015). Here, to better understand the functions of histone H3 methylation in *B. cinerea*, we identified a K9 histone H3 methyltransferase gene *BcDIM5* in *B. cinerea* through Blastp searching the homologs of DIM5 in *N. crassa*. This protein contains a PreSET domain, a SET domain, and a PostSET domain, which is the typical structure of a H3K9 methyltransferase. We characterized the function of *BcDIM5*. The results indicate that *BcDIM5* is required for hyphal growth and the production of conidiophores and sclerotia. Furthermore, pathogenicity assays indicate that BcDIM5 is essential for full virulence of *B. cinerea. BcDIM5* knockout transformants showed down-regulated expression of some pathogenic genes and H3K9 trimethylation was drastically decreased *in vivo*, but two other genes *BcHP1* and *BcDIM2* were not required for the development and virulence of *B. cinerea*. Taken together, these results indicate that BcDIM5 is important for the development and virulence of *B. cinerea*.

## Materials and methods

### Fungal strains and culture conditions

*B. cinerea* wild-type strain B05.10 was used in this study. The fungus was grown on potato dextrose agar (200 g potato, 20 g glucose per liter, 2% agar), and incubated for 7–30 days at 20°C Knockout transformants were cultured on PDA amended with 75 μg/mL hygromycin B (Calbiochem, San Diego, CA, USA). Complementary transformants were cultured on PDA amended with 75 μg/mL hygromycin B and 50 μg/mL G418. *Escherichia coli* strain DH5α was used to propagate all of the plasmids, and *Agrobacterium tumefaciens* strain EHA105 was used for the transformation of fungi. Seedlings of *Arabidopsis thaliana* (ecotype Columbia-0) were grown in the greenhouse at 20 ± 2°C for 1 month under a 12 h light/dark cycle with 70% relative humidity.

### Gene knockout and complementation by protoplast transformation

*BcDIM5, BcDIM2*, and *BcHP1* were knocked out in the WT strain B05.10 using the hygromycin B-resistance gene (*Hyg*) to replace the partial sequences of *BcDIM5, BcDIM2*, and *BcHP1* (Figure S1). To construct the Δ*BcDIM5* disruption vector, a 753-bp DNA fragment named 3U with the *Eco*RI restriction sites at 5′- and 3′-terminus and a 740-bp DNA fragment named 3D with the *Eco*RI restriction sites at 5′- and 3′-terminus were PCR-amplified from genomic DNA of strain B05.10. Using pKS1004 vector as the template, the 740-bp HY fragments were amplified by primers HYG-F and HY-R, and the 1133-bp YG fragments were amplified by primers YG-F and HYG-R. The 3U and HY fragments were fused with PCR to obtain a 1465-bp fragment named 3UHY, and 3D and YG fragments were fused with PCR to obtain a 1936-bp fragment named 3DYG. After digestion with enzymes *Eco*RI, the DNA fragments 3UHY and 3DYG were separately collected and inserted into the linearized plasmid pBluescriptII KS1004 for sequencing. 3UHY and 3DYG were individually linearized and transformed separately into the protoplasts of *B. cinerea* wild type strain B05.10 using the PEG-mediated protoplast transformation technique (Wei et al., 2013). For complementation assays, the 5.75-kb PCR product containing a 3-kb upstream sequence (which contained the endogenous promoter), a full-length *BcDIM5* gene coding region, and a 1-kb downstream sequence was amplified from the wild type strain B05.10 genomic DNA using primers DIM-5-CF-*Bam*HI and DIM-5-CR-*Bam*HI and cloned into the same sites of p3300neoIII to generate the *BcDIM5* complementary vector p3300neoIII-*BcDIM5*, which was then transformed into *BcDIM5* knockout transformants using the *A. tumefaciens*-mediated transformation technique.

### DNA extraction and southern blot analysis

The wild-type strain and knockout transformants were cultured on PDA plates covered with cellophane membranes, and mycelia were harvested at 3 days post inoculation (dpi). Genomic DNA was extracted using the CTAB method. Southern blot analysis was conducted following the method described by Wei et al. (2016). The genomic DNA of 15 μg was completely digested with *Hin*dIII, separated by electrophoresis on 0.8% agarose gel and transferred onto a Hybond N+ membrane (Amersham Pharmacia Biotech). The probe was amplified the specific fragment of hygromycin *hph* gene and be labeled by DIG (GE healthcare). The nylon membranes were autoradiographed and analyzed using Bio-imaging analyser BAS-1800II (FUJIFILM, Tokyo, Japan).

### Pathogenicity tests

Pathogenicity tests of transformed and wild-type *B. cinerea* strains were performed on Arabidopsis, tomato, soybean by the inoculation of detached leaves with young non-sporulating mycelium or conidial suspensions. Leaves were harvested from 4-week-old plants and placed in a transparent plastic box lined with tissue moistened with sterile water. Leaves were inoculated with 2-mm-diameter plugs of 3-day-old mycelium. Alternatively, conidia were collected from 10-day-old plates and suspended in water to a final concentration of 5 × 10^5^ conidia/mL. Droplets of 5 μL were applied to the leaves. Storage boxes containing inoculated leaves were incubated in a growth cabinet at 20°C with 16 h of daylight. Disease development on leaves was recorded daily as the radial spread from the inoculation point to the lesion margin. Pathogenicity assays on leaves were repeated three times using at least three leaves per assay.

### Quantitative real-time PCR

The mRNA transcripts were measured using a SYBR Green I real-time PCR assay in a CFX96 real-time PCR detection systems (Applied Biosystems). The thermal cycling conditions were 95°C for 2 min for predegeneration, 40 cycles of 95°C for 20 s for denaturation, 60°C for 20 s for annealing and 72°C for 20 s for extension. All of the reactions were run in triplicate by monitoring the dissociation curve to control the dimers. The *B. cinerea* β -tubulin gene was used as reference gene. qRT-PCR was used to examine the expression of *B. cinerea Bcsod1* (BC1G_00558), *BcSpl1* (BC1G_02163), *BcVeA* (BC1G_02976), *BcBOT2* (BC1G_06357), *Bcmp3* (BC1G_07144), *BcVELB* (BC1G_11858), *BcPKS* (BC1G_15837), *Bcbot1* (BC1G_16381)*, BcDIM2* (*BC1G_12419*), *BcHP1* (*BC1G_06432*), and *BcDIM5* (*BC1G_11188*). The primers for qRT-PCR are listed in Table S1.

### Western blot analysis of H3K9 trimethylates

Mycelia were harvested and ground in liquid nitrogen. Two hundred milligrams powders were resuspended in 400 μL cell lysis buffer on ice (Beyotime, China). Total proteins of mycelium extract were centrifuged at 12,000 rpm at 4°C for 10 min, and the supernatant was used for western-blot analysis. Proteins were separated by 12% PAGE and electroblotted to a nitrocellulose membrane at 25 V for 40 min. The membrane was blocked with Tris-buffered saline plus Tween 20 containing 5% skim milk powder for 2 h at room temperature. After incubation with primary antibody and then with secondary antibody, the membrane was transferred for protein detection using a Thermo SuperSignal West Pico kit (Thermo Scientific). The sources and dilutions of antibodies were as follows: Histone H3 monoclonal antibody (Abmart, at 1: 2000), Histone H3 tri methyl K9 monoclonal antibody (Abmart, at 1: 2000), and goat anti-mouse horseradish peroxidase-conjugated antibody (Abmart, at 1: 5000).

## Results

### *BC1G_11188* was predicted as a histone H3 lysine 9 methyltransferase

**Figure 1:**
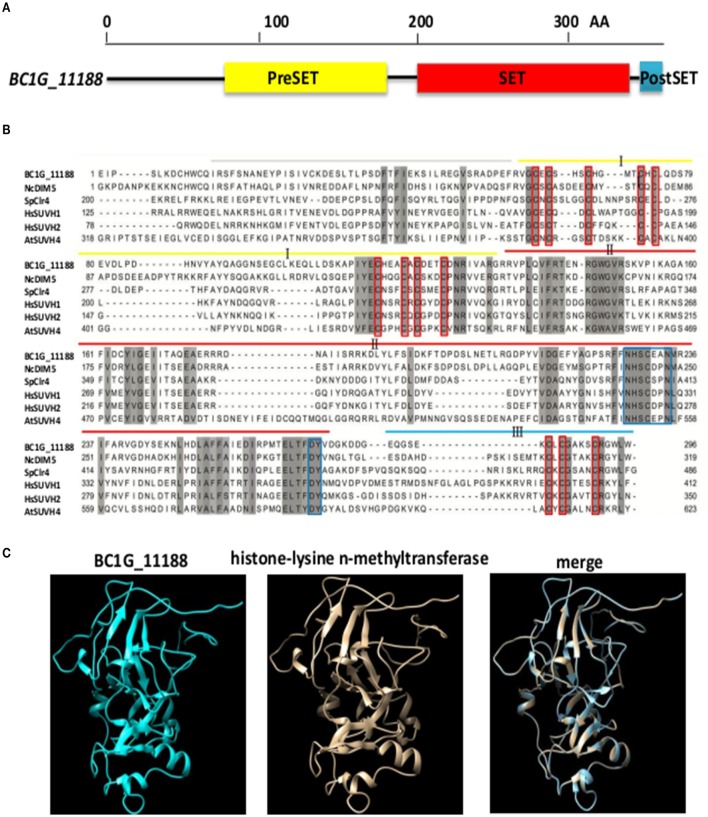
***BcDIM5* was predicted as a H3K9 methyltransferase in *B. cinerea*. (A)** Schematic illustrations of *BcDIM5*. The conserved domains were predicted by the SMART web site. **(B)** Multiple sequence alignment of H3K9 methyltransferase. Comparison of the DIM5 from *Botrytis cinerea* (*BcDIM5*), *Neurospora crassa* (*NcDIM5*), *Schizosaccharomyces pombe* (*SpClr4), Homo sapiens* (*HsSUVH1* and *HsSUVH2*), and *Arabidopsis thaliana* (*AtSUVH4*). The more similar the amino acids are, the darker the background is. **(C)** Representation of the three-dimensional structure of H3K9 methyltransferase and predicted *BcDIM5*. Total residues were modeled with 100% confidence by the single highest scoring template H3K9 methyltransferase (c4qeoA) in the Phyre database.

The *B. cinerea BC1G_11188* gene (GenBank accession: XM_001550366.1) is a single copy gene consisting of 4 exons and 3 introns and encoding a peptide of 357 amino acid residues. The protein contains the typical domain structures of H3K9 methyltransferase (Figure 1A), including a PreSET domain, a SET domain, and a PostSET domain (Rea et al., 2000; Tamaru and Selker, 2001; Adhvaryu et al., 2005). SET domain contains two conserved sequences of NHXCXPN and DY, which can form an AdoMet binding site and the catalytic activity site of methyl transferase, and participates in the formation of DIM5 hydrophobic structure (Zhang et al., 2002). PreSET domain contains nine conserved cysteine residues that can be combined with three zinc ions to form a zinc cluster. PostSET domain contains three conserved cysteine residues involved in the binding of DIM5 to AdoMet (Figure 1B). Similar to the histone lysine methyltransferases of NcDIM5, SpClr4, HsSUVH1, HsSUVH2, and AtSUVH4, BC1G_11188 includes cysteine-rich sequences that flank a SET domain (Figure 1B). The results of Bioinformatics Toolkit HHpred analysis indicate that the structure of *BC1G_11188* is similar to the three-dimensional structure of histone lysine n-methyltransferase (Template: c4qeoA, Confidence: 100%; Figure 1C). In summary, the protein coded by *BC1G_11188* resembles histone H3 lysine 9 methyltransferase. Thus, we designated this gene which was derived from the homologs of *DIM5* in *N. crassa* as “*BcDIM5*.”

### Generation of *BcDIM5* knockout and complementary transformants

To examine the function of *BcDIM5*, the *BcDIM5* knockout transformants were generated through homologous recombination of the *BcDIM5* open reading frame with a gene conferring hygromycin resistance (HYG) (Figure S1A). Positive knockout transformants were amplified with PCR (Figure S1B), and three *BcDIM5* knockout transformants (21, 24, and 25) were identified. Reverse transcription PCR was employed to detect the *BcDIM5* gene expression. The results showed that there were no transcripts in the three knockout transformants (Figure S1C). Two transformants (Δ*BcDIM5-21* and Δ*BcDIM5-24*) were further confirmed by Southern blot analysis (Figure S1D). The mutant Δ*BcDIM5-21* was used for phenotypic analysis and complemented with the full-length gene of wild type*BcDIM5*. After being verified by PCR, the complemented strain *BcDIM5-C1* was chosen for further study (Figure S1E).

### *BcDIM5* played an important role in *B. cinerea* growth and development

**Figure 2:**
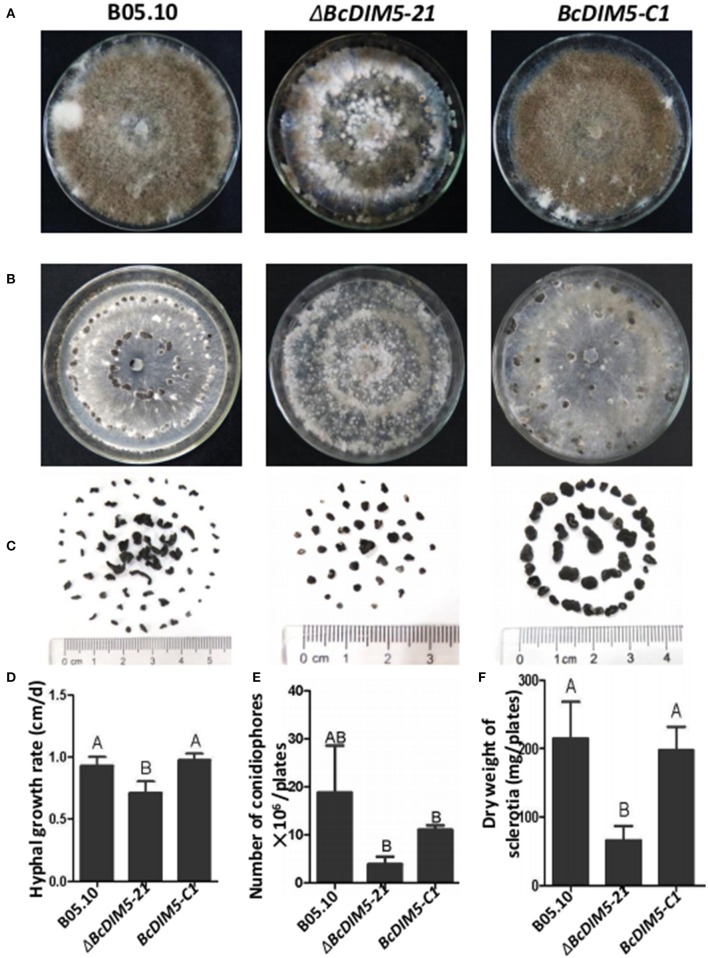
**Biological characterization of the wild-type strain, *BcDIM5* knockout, and complemented transformants**. **(A)** Comparison of the phenotypes of the *BcDIM5* knockout and complemented strain. All the strains were grown on a PDA plate at 20°C for 15 days under a 12 h light/12 h dark photoperiod. **(B)** Comparison of the phenotypes of the *BcDIM5* knockout and complemented strain. All the strains were grown on a PDA plate at 20°C for 15 days under dark condition. **(C)** Sclerotia produced by strains on a PDA plate at 20°C for 30 days under dark condition. **(D)** Comparison of the hyphal growth rate of the *BcDIM5* knockout and complemented strains. Three independent replications were performed. Bars indicate the standard error. The values are presented as the mean ± s.d. Differentiation was evaluated by *t*-test. Different letters on a graph indicate significant differences, *P* < 0.05. **(E)** Comparison of the number of conidiophores per plate of *BcDIM5* knockout and complemented strains. Three independent replications were performed, *P* < 0.05. **(F)** Comparison of dry weight of sclerotia produced by *BcDIM5* knockout and complemented strains. Three independent replications were performed, *P* < 0.05.

To determine the role of *BcDIM5* in the growth and development of *B. cinerea*, we compared the hyphal growth rate, the number of conidiophores and dry weight of sclerotia of the wild-type strain, Δ*BcDIM5-21* strain and the *BcDIM5-C1* complemented strain. Although *BcDIM5* did not affect the morphology of tip hyphae (Figure S2), the hyphal growth rate was significantly reduced in Δ*BcDIM5-21* strains, while this phenotype was restored after complementation (Figure 2D). *BcDIM5* affected the development of conidiophores and sclerotia, and the wild-type strain could averagely produce 2 × 10^7^ conidiophores per plate, while Δ*BcDIM5*-21 strains only produced 3 × 10^6^ conidiophores per plate, and the conidiophore production of *BcDIM5*-C1 complemented strain was restored to 1.1 × 10^7^ conidiophores per plate (Figures 2A,E). Although Δ*BcDIM5-21* strain was able to produce conidiophores, the time required for conidiophore initiation was about 3–4 days longer than that for the normal strain. The dry weight of sclerotia produced by wild-type *B. cinerea* was 217 mg per plate, while Δ*BcDIM5-21* strains only produced 57 mg sclerotia per plate, and the sclerotia was small and round. The sclerotia production of *BcDIM5-C1* complemented strain was restored to 200 mg per plate, which was similar to that of the wild type (Figures 2B,C,F). These data clearly suggest that *BcDIM5* knockout has a deleterious effect on hyphal growth and the development of conidiophores and sclerotia, meanwhile, the reduced hyphal growth rate of ΔBcDIM5-21 strains may delay conidiophore development and the sclerotia formation.

### *BcDIM5* knockout transformants exhibited decreased virulence

**Figure 3:**
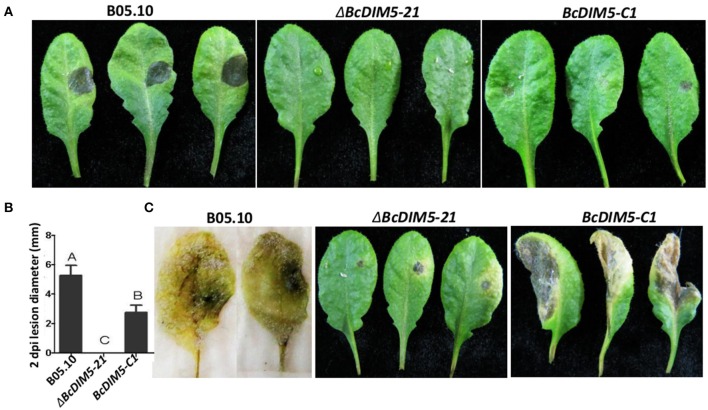
**Pathogenicity assays of the Δ*BcDIM5-21* strain of *B. cinerea* on detached Arabidopsis leaves. (A)** Four-week-old Arabidopsis detached leaves were inoculated by 5 μL conidia (concentration is 5 × 10^5^ spores/mL) of wild type B05.10 (left), *BcDIM5* knockout Δ*BcDIM5-21* strain (middle), *BcDIM5* complemented *BcDIM5-C1* strain (right). The representative symptoms were photographed at 2 dpi. The experiment was repeated three times. **(B)** Comparison of the lesion diameters of the wild-type B05.10 strain, *BcDIM5* knockout, and complemented strains. **(C)** Four-week-old Arabidopsis detached leaves were inoculated by 5 μL conidia (concentration is 5 × 10^5^ spores/mL) of wild-type B05.10 strain (left), *BcDIM5* knockout Δ*BcDIM5-21* strain (middle), *BcDIM5* complemented *BcDIM5-C1* strain (right). The representative symptoms were photographed at 5 dpi after inoculation.

To determine the role of *BcDIM5* in pathogenicity, detached Arabidopsis leaves were inoculated with conidia of the wild-type strain, Δ*BcDIM5-21* strain and *BcDIM5-C1* complemented strain. At 2 dpi, the inoculation of the wild-type strain B05.10 caused the formation of necrotic lesions with 5–6 mm diameter, and the leaves completely rotted away at 5 dpi (Figure 3). However, the leaves inoculated with the Δ*BcDIM5-21* strains exhibited no necrotic lesions at 2 dpi, and only slight necrotic lesions in the infection site at 5 dpi. The pathogenicity of the *BcDIM5-C1* complemented strain was only partially restored, as the diameter of the necrotic lesions was half of that induced by wild-type strain (Figure 3). We further used mycelium pieces to inoculate soybean and tomato leaves. Similar results were observed in Δ*BcDIM5-21* and Δ*BcDIM5-24* mutants. The virulence of the *BcDIM5* knockout transformants was significantly reduced, and only small lesions were observed on the detached leaves of soybean and tomato (Figure S3), indicating that the virulence reduction is not host species-specific. These results indicate that *BcDIM5* plays crucial roles in the virulence.

### Pathogenic genes were down-regulated in *BcDIM5* knockout transformants

**Figure 4:**
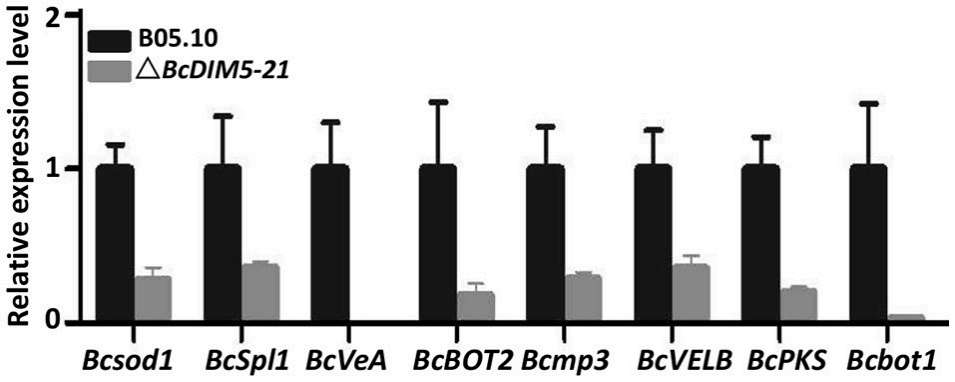
**Expression of the genes associated with pathogenesis in wild-type B05.10 strain and BcDIM5 knockout Δ*BcDIM5-21* strains**. *Botrytis cinerea* Bcsod1 (BC1G_00558), BcSpl1 (BC1G_02163), BcVeA (BC1G_02976), BcBOT2 (BC1G_06357), Bcmp3 (BC1G_07144), BcVELB (BC1G_11858), BcPKS (BC1G_15837), Bcbot1 (BC1G_16381).

To determine whether *BcDIM5* regulates the expression of genes associated with pathogenesis, qRT-PCR was performed to analyze the expression of eight genes involved in the pathogenic process of *B. cinerea* based on previous studies. These eight genes included a Cu-Zn-superoxide dismutase gene *Bcsod1*, which is slightly increased by 0.5 mM H_2_O_2_ and significantly increased by 2.0 mM H_2_O_2_ (Rolke et al., 2004); a cerato-platanin family protein gene *BcSpl1*, which was induced in tobacco systemic resistance to two plant pathogens (Frías et al., 2013); two velvet-like genes *BcVEA* and *BcVELB*, which act as negative regulators for conidiation and melanin biosynthesis in *B. cinerea* (Yang et al., 2013); a presilphiperfolan-8 beta-alpha synthase gene *BcBOT2*, which encode the sesquiterpene synthase that is responsible for the committed step in the biosynthesis of botrydial (Wang et al., 2009); a mitogen-activated protein kinase gene *Bcmp3*, which is required for normal saprotrophic growth, conidiation, plant surface sensing and host tissue colonization (Rui and Hahn, 2007), a polyketide synthase gene *BcPKS* (Dalmais et al., 2011) and a cytochrome P450 monooxygenase gene *Bcbot1* (Siewers et al., 2005). Expression analyses revealed that all of these eight genes were significantly down-regulated in the *BcDIM5* knockout transformants compared with in the wild type (Figure 4). These data indicate that many pathogenic genes may be regulated by *BcDIM5*.

### H3K9 trimethylation level was drastically decreased in *BcDIM5* knockout transformants

**Figure 5:**
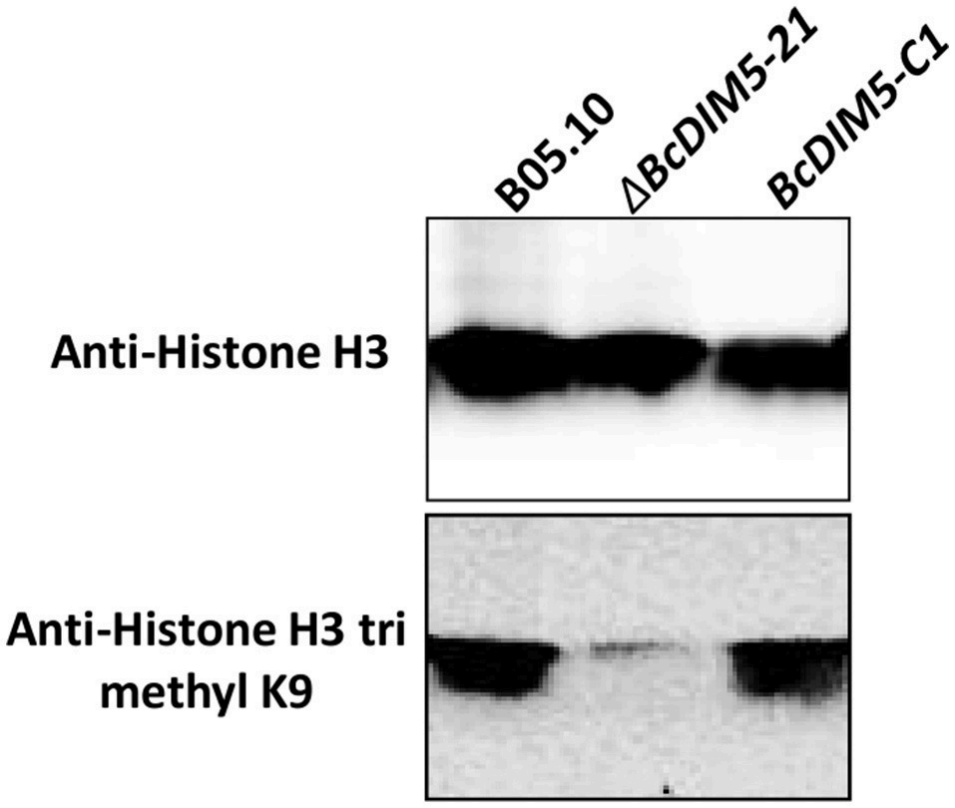
**Western-blot analysis of *B. cinerea* using antibodies histone H3 or trimethylated H3K9**. In each case, 100 μg total proteins from wild-type B05.10, Δ*BcDIM5-21*, or *BcDIM5-C1* strains were fractionated by SDS–PAGE (12%) and analyzed using the indicated antibodies. The monoclonal anti-Histone H3 was used as internal loading reference.

To investigate the possible effects of DIM5 on the trimethylation of H3K9, we carried out western-blot to analyze the histone methylation level of the wild-type strain, Δ*BcDIM5-21* and *BcDIM5-C1* (Figure 5). All the strains presented equally robust signals when detected using the antibody histone H3. Notably, trimethylated H3K9 was detectable in bulk from the wild-type strain and *BcDIM5-C1*, while knockout transformant Δ*BcDIM5-21* extinguished the trimethylated H3K9 signal, suggesting that BcDIM5 is dominantly responsible for the trimethylation of H3K9, and DIM5 may generate trimethylated H3K9 from unmodified histone H3 *in vivo*.

### *BcDIM2* and *BcHP1* were not required for virulence in *B. cinerea*

**Figure 6:**
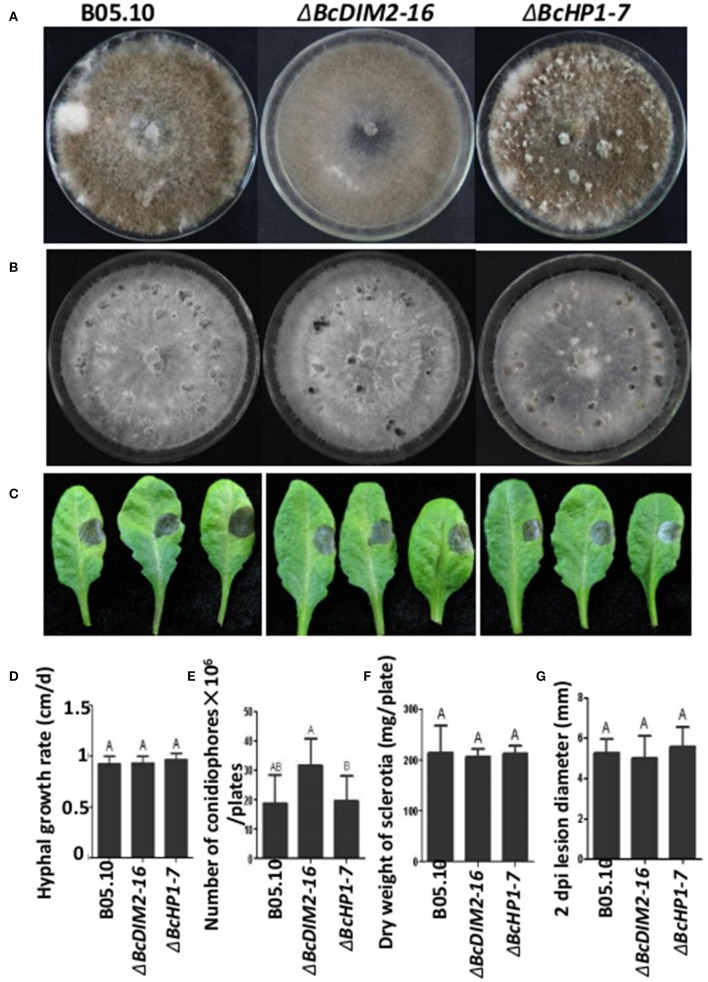
**Phenotypes of *BcDIM2* and *BcHP1* knockout transformants. (A)** Comparison of the phenotypes of the Δ*BcDIM2-16* and Δ*BcHP1-7* knockout transformants. All the strains were grown on a PDA plate at 20°C for 15 days under a 12 h light/12 h dark photoperiod. **(B)** Comparison of the phenotypes of the Δ*BcDIM2-16* and Δ*BcHP1-7* knockout transformants. All the strains were grown on a PDA plate at 20°C for 15 days under dark condition. **(C)** Pathogenicity assays of the Δ*BcDIM2-16* and Δ*BcHP1-7* knockout transformants of *B. cinerea* on detached Arabidopsis leaves. **(D)** Comparison of the hyphal growth rate of the Δ*BcDIM2-16* and Δ*BcHP1-7* knockout transformants. **(E)** Comparison of the number of conidiophores per plate of Δ*BcDIM2-16* and Δ*BcHP1-7* knockout mutants. **(F)** Comparison of dry weight of sclerotia produced by Δ*BcDIM2-16* and Δ*BcHP1-7* knockout transformants. **(G)** Comparison of the lesion diameters of the wild type, Δ*BcDIM2-16* and Δ*BcHP1-7* knockout transformants.

In *N. crassa*, DIM5 trimethylates H3K9, and H3K9me3 directs DNA methylation through a complex containing heterochromatin protein 1 (HP1) and the DNA methyltransferase DIM2. We also investigated whether DIM2 and HP1 play important roles in *B. cinerea* development and virulence. Based on the homology to *N. crassa DIM2* and *HP1*, we cloned *BcDIM2* (*BC1G_12419*) and *BcHP1* (*BC1G_06432*) from *B. cinerea*. *BcDIM2* contains typical domain structures of DNA methyltransferase and *BcHP1* contains typical domain structures of CD (chromo domain) and CSD (chromo shadow domain; Paro and Hogness, 1991; Aasland and Stewart, 1995). RT–PCR analysis showed that *BcDIM5, BcDIM2*, and *BcHP1* were all expressed in mycelium growth, sclerotial development and infection stages (Figure S4). *BcDIM5* was constitutively expressed in these three stages, and *BcDIM2* was significantly up-regulated during the infection stage, while *BcHP1* was significantly down-regulated during the sclerotial development. Through homologous recombination methods, we got two knockout transformants Δ*BcDIM2-16* and Δ*BcHP1-7*. There were no significant changes in the phenotype and number of produced conidiophores as well as in hyphal growth rate, dry weight of sclerotia and virulence among the wild-type, Δ*BcDIM2-16* and Δ*BcHP1-7* strains (Figure 6). Therefore, it can be concluded that BcDIM5 is required for the development and virulence whereas BcDIM2 and BcHP1 are not required for virulence in *B. cinerea*.

## Discussion

Epigenetic alterations have important roles in certain biological processes such as cell phenotypic conversion, cell proliferation, and cell death by regulating transcriptional activity (Reik, 2007; Papp and Plath, 2011). The effect of epigenetic processes on transcription depends on various post-translational histone modifications (Wierda et al., 2015). The major histone modifications include acetylation, phosphorylation, methylation, and ubiquitylation (Arnaudo and Garcia, 2013). In this paper, we further described the extended role of H3K9 methyltransferase *BcDIM5* in *B. cinere. BcDIM5* was predicted as a methyltransferase with a SET domain (Figure 1). Although there is no evidence to show that BcDIM5 has methyltransferase activity *in vitro*, the structure-guided sequence alignment indicates that *BcDIM5* has all of the methyltransferase characteristics, since all the aligned sequences were verified to be active methyltransferases previously (Zhang et al., 2002). In addition, as shown in Figure 5, western blot analysis using dimethyl H3K9 antibodies demonstrates that trimethylated H3K9 in *BcDIM5* knockout transformants was drastically decreased and the phenotypes of the transformants were restored after complementation. These findings suggest that *BcDIM5* has methyltransferase activity *in vivo*.

Analyses of the Δ*BcDIM5* and complemented Δ*BcDIM5* strains in this study indicate that Δ*BcDIM5* is involved in sclerotia production. The Δ*BcDIM5* transformants exhibited declined hyphal growth rate and reduced production of conidiophores and sclerotia (Figure 2). Declined growth rate of Δ*BcDIM5* strians may affect the conidiophores development and sclerotia formation. In order to avoid this, we delayed the statistical time for development of conidiophores and sclerotia for 15 or 30 days, respectively. The results suggest that *BcDIM5* is required for normal hyphal growth, conidiation, and sclerotia development. Additionally, in the pathogenicity assays, Δ*BcDIM5* knockout transformants exhibited drastic reduction in virulence compared with wild type B05.10, while complemented Δ*BcDIM5* strain could partially restore the phenotype (Figure 3). These results indicate that BcDIM5 plays a crucial role in the development and pathogenicity of *B. cinerea*, which is consistent with the results of previous studies. In histone methyltransferase G9a knockout mice, H3K9 methylation was drastically reduced, resulting in severe growth retardation and early lethality, which indicates that G9a is essential for early mouse embryo development (Tachibana et al., 2002, 2005). We then identified that eight pathogenic genes expression in *BcDIM5* knockout transformants (Figure 4). *Bcsod1*, which is increased by H_2_O_2_ accumulation, is drastically declined in Δ*BcDIM5* strain. *Bcmp3*, which is required for normal saprotrophic growth, is drastically declined in Δ*BcDIM5* strain. *BcSpl1*, which is induced in systemic resistance, is drastically declined in Δ*BcDIM5* strain. *BcVeA* and *BcVELB* are involved in the regulation of fungal development, oxidative stress response and virulence in *B. cinerea*, are drastically declined in Δ*BcDIM5* strain. *BcBOT1, BcBOT2*, and *BcPKS*, associated with P450, botrydial and phytotoxin botcinic acid biosynthesis, respectively, are drastically declined in Δ*BcDIM5* strain. Down-regulated eight pathogenic genes may lead to the drastically declined pathogenicity of Δ*BcDIM5* strain. High throughput sequencing can identify the suppressed genes in Δ*BcDIM5* strain, and the transcriptionally suppressive function of BcDIM5 may depend on its histone methyltransferase activity, but the molecular mechanism needs further investigation.

In *N. crassa*, all DNA methylation requires the DNA methyltransferase DIM2 (Kouzminova and Selker, 2001). DNA methylation also requires the histone H3K9 methyltransferase DIM5 (Tamaru and Selker, 2001; Tamaru et al., 2003) and heterochromatin protein HP1 (Freitag et al., 2004). H3K9 trimethylation directs DNA methylation through a complex containing HP1 and the DNA methyltransferase DIM2 (Honda et al., 2012). Based on the homology to *N. crassa DIM2* and *HP1*, we cloned *BcDIM2* and *BcHP1*. The Δ*BcDIM2-16* and Δ*BcHP1-7* knockout transformants showed no significant change in the development of conidiophores and sclerotia and virulence compared with wild type B05.10 (Figure 6). These results indicate that DNA methylation does not play important roles in this process. This growth phenotype is in agreement with the results of knockout of *N. crassa DIM2* and *Aspergillus nidulans dmtA* (DNA methyltransferase homolog A), which did not affect the normal growth of the strain (Kouzminova and Selker, 2001; Lee et al., 2008). Although, DNA methylation is common in organisms such as bacteria, fungi, higher plants, and mammals, we did not use genomic DNA sequencing to detect the level of DNA methylation in *B. cinerea*, thus it is not known yet whether genomic DNA methylation is missing in Δ*BcDIM2-16* knockout transformants. These data may be needed to explore the function of *BcDIM2* in *B. cinerea*. HP1 plays a key role in heterochromatin formation and gene silencing, and is a positive regulator of active transcription in euchromatin (Kwon and Workman, 2011; Yearim et al., 2015). A number of recent studies have linked HP1 proteins to the DNA damage pathway, and HP1 proteins are independent of H3K9 trimethylation (Ayoub et al., 2008; Luijsterburg et al., 2009). *Neurospora* HP1 is required for normal growth of fungi (Freitag et al., 2004). In *Schizosaccharomyces pombe*, the HP1 homologs swi6 and chp2/clo2 are important for normal growth but are not essential for viability (Halverson et al., 2000). In contrast, Δ*BcHP1-7* knockout transformants in *B. cinerea* did not show noticeable growth defects. Most eukaryotes contain several HP1 homologs that display dramatic differences in both subcellular localizations and functions (Ryu et al., 2014). The function of *BcHP1* in *B. cinerea* needs further research. Unlike that of *BcDIM2* and *BcHP1*, knockout mutation of the histone methyltransferase gene *BcDIM5* causes growth abnormality, pathogenic gene silencing and virulence reduction in *B. cinerea*, suggesting that it is histone methylation rather than DNA methylation that is involved in the development and virulence of *B. cinerea*. The detailed mechanisms of *BcDIM5* to regulate the growth and pathogenic gene silencing need further investigation.

## Author contributions

XZ, XL contributed equally to the article, XZ, XL, JC, and TC designed the research and wrote the paper; XZ, XL, YZ, and TC executed the experiments. XZ, XL, YZ, JC, JX, YF, DJ, and TC performed the data and analyses. All authors read and approved the final manuscript.

### Conflict of interest statement

The authors declare that the research was conducted in the absence of any commercial or financial relationships that could be construed as a potential conflict of interest.

## Supplementary material

Figure S1 **Identification of *BcDIM5* knockout and complemented transformants. (A)** Principle of homologous recombination. **(B)** *BcDIM5* knockout transformants identified by PCR. **(C)** *BcDIM5* knockout transformants identified by RT-PCR. **(D)** *BcDIM5* knockout transformants identified by Southern blot using *HYG* labeled with DIG as probe. **(E)** *BcDIM5* complemented transformants identified by PCR.

Figure S2 **Morphological comparison of the hyphal tips of *BcDIM5* knockout and complemented transformants**.

Figure S3 **Virulence assay of *BcDIM5* knockout and complemented transformants on detached soybean and tomato leaves**. Virulence was evaluated based on the lesion diameter at 20°C for 48 h. **(A)** Virulence assay of *BcDIM5* knockout and complemented transformants on detached soybean leaves. **(B)** Virulence was evaluated based on the lesion diameter on soybean leaves. **(C)** Virulence assay of *BcDIM5* knockout and complemented transformants on tomato leaves. **(D)** Virulence was evaluated based on the lesion diameter on tomato leaves.

Figure S4 **Expression level of genes in different phase**. Forty-eight hour indicates the wild type strain B05.10 grown in PDA for 48 h, which is the hyphal growth stage; 156 h indicates the wild-type strain B05.10 grown in PDA for 156 h, which is the sclerotial development stage. IN indicates the wild-type strain B05.10 inoculated on *A. thaliana* leaves for 48 h, which is the infection stage.

Table S1 **Primers used in the study**.

## References

[B1] Aasland R. Stewart A. F. (1995). The chromo shadow domain, a second chromo domain in heterochromatin-binding protein 1, HP1. Nucleic Acids Res. 23, 3168–3173. 10.1093/nar/23.16.3168 7667093 PMC307174

[B2] Adhvaryu K. K. Morris S. A. Strahl B. D. Selker E. U. (2005). Methylation of histone H3 lysine 36 is required for normal development in *Neurospora crassa*. Eukaryotic Cell 4, 1455–1464. 10.1128/EC.4.8.1455-1464.2005 16087750 PMC1214527

[B3] Arnaudo A. M. Garcia B. A. (2013). Proteomic characterization of novel histone post-translational modifications. Epigenetics Chromatin 6:24. 10.1186/1756-8935-6-24 23916056 PMC3737111

[B4] Ayoub N. Jeyasekharan A. D. Bernal J. A. Venkitaraman A. R. (2008). HP1-beta mobilization promotes chromatin changes that initiate the DNA damage response. Nature 453, 682-U614. 10.1038/nature06875 18438399

[B5] Bedford M. T. Clarke S. G. (2009). Protein arginine methylation in mammals: who, what, and why. Mol. Cell 33, 1–13. 10.1016/j.molcel.2008.12.013 19150423 PMC3372459

[B6] Berger S. L. (2007). The complex language of chromatin regulation during transcription. Nature 447, 407–412. 10.1038/nature05915 17522673

[B7] Choquer M. Fournier E. Kunz C. Levis C. Pradier J. M. Simon A.. (2007). *Botrytis cinerea* virulence factors: new insights into a necrotrophic and polyphageous pathogen. FEMS Microbiol. Lett. 277, 1–10. 10.1111/j.1574-6968.2007.00930.x 17986079

[B8] Dalmais B. Schumacher J. Moraga J. LE Pêcheur P. Tudzynski B. Collado I. G.. (2011). The *Botrytis cinerea* phytotoxin botcinic acid requires two polyketide synthases for production and has a redundant role in virulence with botrydial. Mol. Plant Pathol. 12, 564–579. 10.1111/j.1364-3703.2010.00692.x 21722295 PMC6640383

[B9] Freitag M. Hickey P. C. Khlafallah T. K. Read N. D. Selker E. U. (2004). HP1 is essential for DNA methylation in neurospora. Mol. Cell 13, 427–434. 10.1016/S1097-2765(04)00024-3 14967149

[B10] Frías M. Brito N. González C. (2013). The *Botrytis cinerea* cerato-platanin BcSpl1 is a potent inducer of systemic acquired resistance (SAR) in tobacco and generates a wave of salicylic acid expanding from the site of application. Mol. Plant Pathol. 14, 191–196. 10.1111/j.1364-3703.2012.00842.x 23072280 PMC6638659

[B11] Halverson D. Gutkin G. Clarke L. (2000). A novel member of the Swi6p family of fission yeast chromo domain-containing proteins associates with the centromere *in vivo* and affects chromosome segregation. Mol. Gen. Genet. 264, 492–505. 10.1007/s004380000338 11129054

[B12] Honda S. Lewis Z. A. Shimada K. Fischle W. Sack R. Selker E. U. (2012). Heterochromatin protein 1 forms distinct complexes to direct histone deacetylation and DNA methylation. Nat. Struct. Mol. Biol. 19, S471. 10.1038/nsmb.2274 22504884 PMC4957970

[B13] Kouzminova E. Selker E. U. (2001). dim-2 encodes a DNA methyltransferase responsible for all known cytosine methylation in Neurospora. EMBO J. 20, 4309–4323. 10.1093/emboj/20.15.4309 11483533 PMC149169

[B14] Kwon S. H. Workman J. L. (2011). The changing faces of HP1: from heterochromatin formation and gene silencing to euchromatic gene expression: HP1 acts as a positive regulator of transcription. Bioessays 33, 280–289. 10.1002/bies.201000138 21271610

[B15] Lan F. Shi Y. (2009). Epigenetic regulation: methylation of histone and non-histone proteins. Sci. China C Life Sci. 52, 311–322. 10.1007/s11427-009-0054-z 19381457

[B16] Lee D. W. Freitag M. Selker E. U. Aramayo R. (2008). A cytosine methyltransferase homologue is essential for sexual development in *Aspergillus nidulans*. PLoS ONE 3:e2531. 10.1371/journal.pone.0002531 18575630 PMC2432034

[B17] Liu C. Lu F. Cui X. Cao X. (2010). Histone methylation in higher plants. Ann. Rev. Plant Biol. 64, 395–420. 10.1146/annurev.arplant.043008.091939 20192747

[B18] Luijsterburg M. S. Dinant C. Lans H. Stap J. Wiernasz E. Lagerwerf S.. (2009). Heterochromatin protein 1 is recruited to various types of DNA damage. J. Cell Biol. 185, 577–586. 10.1083/jcb.200810035 19451271 PMC2711568

[B19] Nakayama J. Rice J. C. Strahl B. D. Allis C. D. Grewal S. I. (2001). Role of histone H3 lysine 9 methylation in epigenetic control of heterochromatin assembly. Science 292, 110–113. 10.1126/science.1060118 11283354

[B20] O'Carroll D. Scherthan H. Peters A. H. Opravil S. Haynes A. R. Laible G.. (2000). Isolation and characterization of Suv39h2, a second histone H3 methyltransferase gene that displays testis-specific expression. Mol. Cell. Biol. 20, 9423–9433. 10.1128/MCB.20.24.9423-9433.2000 11094092 PMC102198

[B21] Papp B. Plath K. (2011). Reprogramming to pluripotency: stepwise resetting of the epigenetic landscape. Cell Res. 21, 486–501. 10.1038/cr.2011.28 21321600 PMC3193418

[B22] Paro R. Hogness D. S. (1991). The Polycomb protein shares a homologous domain with a heterochromatin-associated protein of Drosophila. Proc. Natl. Acad. Sci. U.S.A. 88, 263–267. 10.1073/pnas.88.1.263 1898775 PMC50790

[B23] Rea S. Eisenhaber F. O'Carroll D. Strahl B. D. Sun Z. W. Schmid M.. (2000). Regulation of chromatin structure by site-specific histone H3 methyltransferases. Nature 406, 593–599. 10.1038/35020506 10949293

[B24] Reik W. (2007). Stability and flexibility of epigenetic gene regulation in mammalian development. Nature 447, 425–432. 10.1038/nature05918 17522676

[B25] Rivera C. Saavedra F. Alvarez F. Díaz-Celis C. Ugalde V. Li J.. (2015). Methylation of histone H3 lysine 9 occurs during translation. Nucleic Acids Res. 43, 9097–9106. 10.1093/nar/gkv929 26405197 PMC4627087

[B26] Rolke Y. Liu S. J. Quidde T. Williamson B. Schouten A. Weltring K. M. (2004). Functional analysis of H2O2-generating systems in *Botrytis cinerea*: the major Cu-Zn-superoxide dismutase (BCSOD1) contributes to virulence on French bean, whereas a glucose oxidase (BCGOD1) is dispensable. Mol. Plant Pathol. 5, 17–27. 10.1111/j.1364-3703.2004.00201.x 20565578

[B27] Rui O. Hahn M. (2007). The Slt2-type MAP kinase Bmp3 of *Botrytis cinerea* is required for normal saprotrophic growth, conidiation, plant surface sensing and host tissue colonization. Mol. Plant Pathol. 8, 173–184. 10.1111/j.1364-3703.2007.00383.x 20507489

[B28] Ryu H. W. Lee D. H. Florens L. Swanson S. K. Washburn M. P. Kwon S. H. (2014). Analysis of the heterochromatin protein 1 (HP1) interactome in Drosophila. J. Proteomics 102, 137–147. 10.1016/j.jprot.2014.03.016 24681131

[B29] Siewers V. Viaud M. Jimenez-Teja D. Collado I. G. Gronover C. S. Pradier J. M.. (2005). Functional analysis of the cytochrome P450 monooxygenase gene bcbot1 of *Botrytis cinerea* indicates that botrydial is a strain-specific virulence factor. Mol. Plant Microbe Interact. 18, 602–612. 10.1094/MPMI-18-0602 15986930

[B30] Tachibana M. Sugimoto K. Nozaki M. Ueda J. Ohta T. Ohki M.. (2002). G9a histone methyltransferase plays a dominant role in euchromatic histone H3 lysine 9 methylation and is essential for early embryogenesis. Genes Dev. 16, 1779–1791. 10.1101/gad.989402 12130538 PMC186403

[B31] Tachibana M. Ueda J. Fukuda M. Takeda N. Ohta T. Iwanari H.. (2005). Histone methyltransferases G9a and GLP form heteromeric complexes and are both crucial for methylation of euchromatin at H3-K9. Genes Dev. 19, 815–826. 10.1101/gad.1284005 15774718 PMC1074319

[B32] Tamaru H. Selker E. U. (2001). A histone H3 methyltransferase controls DNA methylation in *Neurospora crassa*. Nature 414, 277–283. 10.1038/35104508 11713521

[B33] Tamaru H. Zhang X. McMillen D. Singh P. B. Nakayama J. Grewal S. I.. (2003). Trimethylated lysine 9 of histone H3 is a mark for DNA methylation in *Neurospora crassa*. Nat. Genet. 34, 75–79. 10.1038/ng1143 12679815

[B34] Tschiersch B. Hofmann A. Krauss V. Dorn R. Korge G. Reuter G. (1994). The protein encoded by the Drosophila position-effect variegation suppressor gene Su(var)3-9 combines domains of antagonistic regulators of homeotic gene complexes. EMBO J. 13, 3822–3831. 7915232 10.1002/j.1460-2075.1994.tb06693.x PMC395295

[B35] Wang C. M. Hopson R. Lin X. Cane D. E. (2009). Biosynthesis of the sesquiterpene botrydial in *Botrytis cinerea*. mechanism and stereochemistry of the enzymatic formation of presilphiperfolan-8 beta-ol. J. Am. Chem. Soc. 131, 8360–8361. 10.1021/ja9021649 19476353 PMC2702122

[B36] Wei W. Zhu W. Cheng J. Xie J. Jiang D. Li G.. (2016). Nox Complex signal and MAPK cascade pathway are cross-linked and essential for pathogenicity and conidiation of mycoparasite Coniothyrium minitans. Sci. Rep. 6:24325. 10.1038/srep24325 27066837 PMC4828707

[B37] Wei W. Zhu W. J. Cheng J. S. Xie J. T. Li B. Jiang D. H.. (2013). CmPEX6, a gene involved in peroxisome biogenesis, is essential for parasitism and conidiation by the sclerotial parasite Coniothyrium minitans. Appl. Environ. Microbiol. 79, 3658–3666. 10.1128/AEM.00375-13 23563946 PMC3675954

[B38] Widom J. (1998). Chromatin structure: linking structure to function with histone H1. Curr. Biol. 8, R788–R791. 10.1016/S0960-9822(07)00500-3 9811600

[B39] Wierda R. J. Rietveld I. M. Van Eggermond M. C. Belien J. A. Van Zwet E. W. Lindeman J. H.. (2015). Global histone H3 lysine 27 triple methylation levels are reduced in vessels with advanced atherosclerotic plaques. Life Sci. 129, 3–9. 10.1016/j.lfs.2014.10.010 25445221

[B40] Williamson B. Tudzynski B. Tudzynski P. Van Kan J. A. (2007). *Botrytis cinerea*: the cause of gray mould disease. Mol. Plant Pathol. 8, 561–580. 10.1111/j.1364-3703.2007.00417.x 20507522

[B41] Xiao B. Jing C. Wilson J. R. Walker P. A. Vasisht N. Kelly G.. (2003). Structure and catalytic mechanism of the human histone methyltransferase SET7/9. Nature 421, 652–656. 10.1038/nature01378 12540855

[B42] Yang Q. Q. Chen Y. F. Ma Z. H. (2013). Involvement of BcVeA and BcVelB in regulating conidiation, pigmentation and virulence in *Botrytis cinerea*. Fungal Genet. Biol. 50, 63–71. 10.1016/j.fgb.2012.10.003 23147398

[B43] Yearim A. Gelfman S. Shayevitch R. Melcer S. Glaich O. Mallm J. P.. (2015). HP1 is involved in regulating the global impact of DNA methylation on alternative splicing. Cell Rep. 10, 1122–1134. 10.1016/j.celrep.2015.01.038 25704815

[B44] Zhang X. Tamaru H. Khan S. I. Horton J. R. Keefe L. J. Selker E. U.. (2002). Structure of the Neurospora SET domain protein DIM-5, a histone H3 lysine methyltransferase. Cell 111, 117–127. 10.1016/S0092-8674(02)00999-6 12372305 PMC2713760

