# Supplementary figures and images for: Histone H3 Lysine 9 Methyltransferase DIM5 Is Required for the Development and Virulence of Botrytis cinerea
Source: Front Microbiol. 2016 Aug 22;7:1289. doi: 10.3389/fmicb.2016.01289 (PMC4992730; doi:10.3389/fmicb.2016.01289)

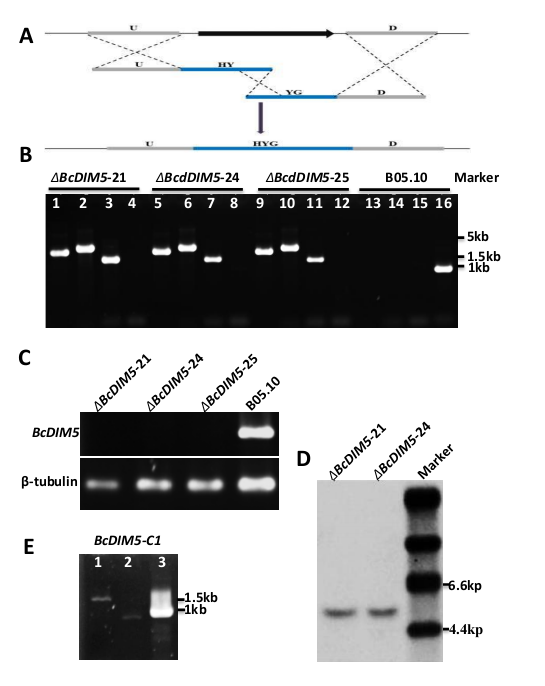

Supplement: Figure S1 — Identification of BcDIM5 knockout and complemented transformants. (A) Principle of homologous recombination. (B) BcDIM5 knockout transformants identified by PCR. (C) BcDIM5 knockout transformants identified by RT-PCR. (D) BcDIM5 knockout transformants identified by Southern blot using HYG labeled with DIG as probe. (E) BcDIM5 complemented transformants identified by PCR. [file Image1.TIF]

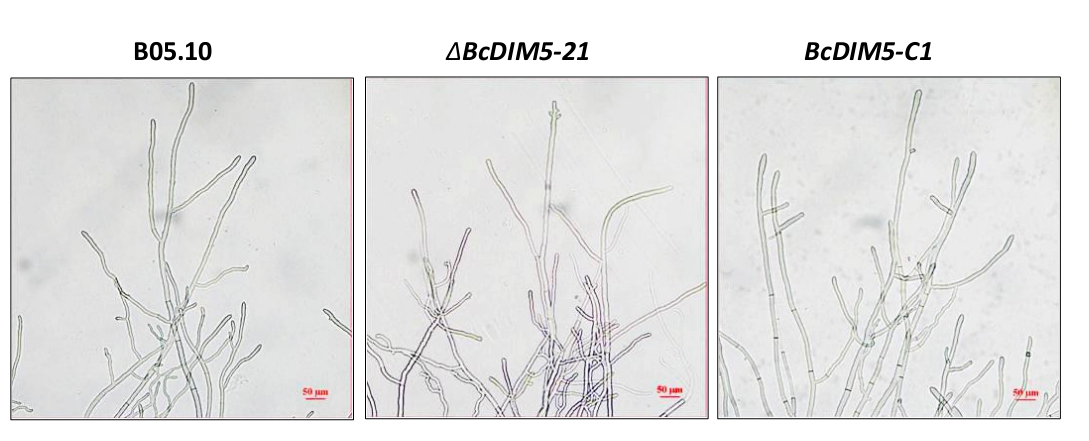

Supplement: Figure S2 — Morphological comparison of the hyphal tips of BcDIM5 knockout and complemented transformants. [file Image2.TIF]

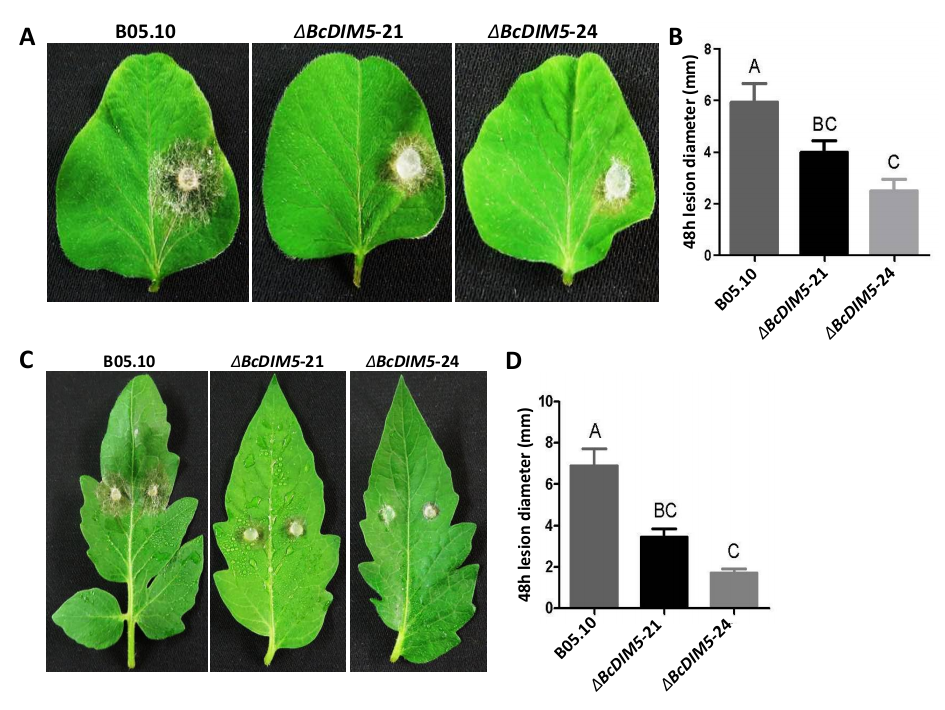

Supplement: Figure S3 — Virulence assay of BcDIM5 knockout and complemented transformants on detached soybean and tomato leaves. Virulence was evaluated based on the lesion diameter at 20°C for 48 h. (A) Virulence assay of BcDIM5 knockout and complemented transformants on detached soybean leaves. (B) Virulence was evaluated based on the lesion diameter on soybean leaves. (C) Virulence assay of BcDIM5 knockout and complemented transformants on tomato leaves. (D) Virulence was evaluated based on the lesion diameter on tomato leaves. [file Image3.TIF]

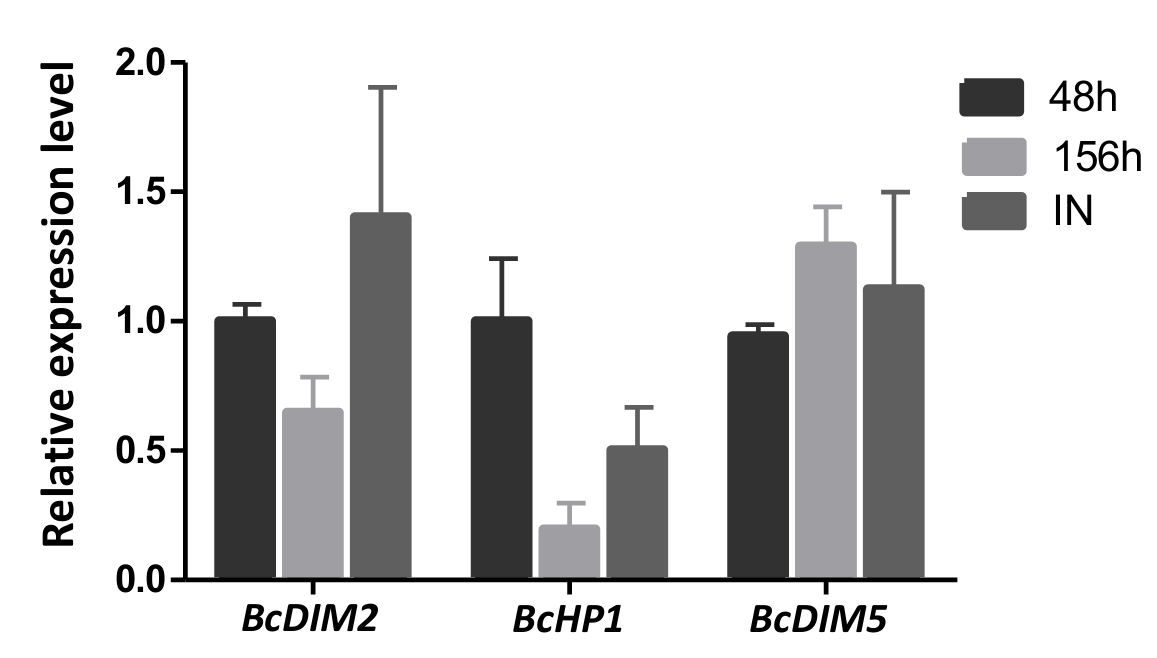

Supplement: Figure S4 — Expression level of genes in different phase. Forty-eight hour indicates the wild type strain B05.10 grown in PDA for 48 h, which is the hyphal growth stage; 156 h indicates the wild-type strain B05.10 grown in PDA for 156 h, which is the sclerotial development stage. IN indicates the wild-type strain B05.10 inoculated on A. thaliana leaves for 48 h, which is the infection stage. [file Image4.TIF]
